# Tumor characteristics, treatments, and survival outcomes in prostate cancer patients with a PSA level < 4 ng/ml: a population-based study

**DOI:** 10.1186/s12885-020-06827-z

**Published:** 2020-04-22

**Authors:** Zhibo Zheng, Zhien Zhou, Weigang Yan, Yi Zhou, Chuyan Chen, Hanzhong Li, Zhigang Ji

**Affiliations:** 1 Department of Urology, Peking Union Medical College Hospital, Peking Union Medical College, Chinese Academy of Medical Sciences, No.1 Shuaifuyuan, Wangfujing, Dong Cheng District, Beijing, 100730 China; 2 Department of International Medical Services, Peking Union Medical College Hospital, Peking Union Medical College, Chinese Academy of Medical Sciences, Beijing, China; 3 Peking Union Medical College, Chinese Academy of Medical Sciences, Beijing, China

**Keywords:** Prostate cancer, PSA < 4 ng/ml, Tumor characteristics, SEER program

## Abstract

**Background:**

To examine the tumor characteristics, treatments and survival outcomes of prostate cancer (PCa) patients with a prostate-specific antigen (PSA) level < 4 ng/ml.

**Methods:**

Of 205,913 men with primary prostate adenocarcinoma in the Surveillance, Epidemiology and End Results (SEER) database (2010 to 2015), 24,054 (11.68%) patients were diagnosed with a PSA level < 4 ng/ml. Comparisons of categorical variables among different groups were performed by using the Chi square test. Multivariate Cox regression analysis was adjusted for age, ethnicity, marital status, insurance status, TNM stage, Gleason grade, treatment and survival. Kaplan-Meier survival curves were constructed for overall mortality and tested by the log-rank test.

**Results:**

PCa patients with a PSA level < 4 ng/ml generally had more favorable tumor characteristics: younger, lower T stage, lower Gleason grade and lower lymph node metastasis rate. However, there were more patients in stage M1 in the group of PSA level < 4 ng/ml than that in the groups of PSA level of 4–10 ng/ml, 10–20 ng/ml and > 20 ng/ml. The multivariate Cox regression model revealed that overall mortality was associated with age, marital status, race, Gleason grade, M stage and treatment approach.

**Conclusions:**

In conclusion, PCa patients with a PSA level < 4 ng/ml have more favorable tumor characteristics at diagnosis and receive more benefit from active treatment. However, those patients with advanced TNM stage and high Gleason grade should be paid more attention in clinical application.

## Background

Prostate cancer (PCa) is the most commonly diagnosed cancer in men worldwide, with an estimated 1.6 million cases and 0.366 million deaths annually [1, 2]. Due to the widespread use of serum prostate-specific antigen (PSA) measurement [3], most PCa patients are now diagnosed with clinically localized disease. PSA is a glycoprotein that is expressed in normal prostate tissue and prostate tumor tissue. Due to lack of basal cells in PCa, the level of PSA is lower in PCa cells than that in normal prostate gland cells, resulting in the disruption of the basement membrane and lumenal structure. Thus, a large number of PSA is released into the circulation [4, 5].

Historical literature suggests that increasing serum PSA level is associated with advanced TNM stage and worse outcomes [6, 7]. Therefore, the pretreatment serum PSA level is an important factor in PCa risk stratification [8]. Several studies have focused on PCa patients with a “normal” PSA level (< 4 ng/ml), who should have better disease characteristics and better outcomes, but were discovered with some biologically aggressive characteristics [9–14]. Doctors may underestimate the aggressiveness of PCa patients under normal PSA level and fail to choose an appropriate treatment method for these patients. Our study aims to use the Surveillance, Epidemiology, and End Results (SEER) database to examine initial tumor characteristics and treatment modalities, and identify prognostic factors in PCa patients with a PSA level < 4 ng/ml.

## Methods

### Data source

The SEER program of the National Cancer Institute collects cancer patient data from population-based cancer registries in several geographic regions in the United States. The SEER database covers approximately 28% of the population in the United States. Available data include patient demographics, tumor characteristics (histology, grading, and TNM stage), treatment and vital status. SEER*Stat 8.3.5 software was used to extract information from the database.

### Study cohort

Data from patients diagnosed with prostate adenocarcinoma (site code C61.9, histological type according to ICD.0.3 code 8140) from January 1, 2010 to December 31, 2015 were extracted from the SEER-18 database with additional treatment fields. PSA information was available in the SEER database beginning in 2017. TNM stage was classified according to the American Joint Committee on Cancer, 6th edition. The following SEER variables were collected: age, year of diagnosis, ethnicity, marital status, insurance status, TNM stage, Gleason score, treatment and survival. Patients with an incomplete pathological diagnosis, a missing Gleason score, missing survival details, multiple primary tumors or missing records for metastatic data were excluded. The PSA value was categorized into the following groups: < 4 ng/ml, 4–10 ng/ml, 10–20 ng/ml and > 20 ng/ml. Treatment methods consisted of radical prostatectomy (RP), brachytherapy (BT) and external beam radiation (EBRT). The treatment approaches were divided into following three groups: no RP or radiotherapy (RT), receiving RP with or without RT and receiving RT without RP.

### Statistical analysis

All the data collected in this study were analyzed by SPSS 25.0 (SPSS Inc., Chicago, IL, USA) and Intercooled Stata SE 15.0 (Stata Corporation, College Station, TX, USA). Demographic data, clinical information and tumor features were summarized with descriptive statistics. Comparisons of categorical variables among different PSA level groups were performed by using the Chi square test. The multivariate Cox proportional hazards model was used to assess the relative impacts of risk factors for cancer-specific survival (CCS) and overall survival (OS) on PCa patients with a PSA level < 4 ng/ml. Kaplan-Meier survival curves were constructed for overall mortality; the differences between the curves were tested by the log-rank test. A two-sided *P* < 0.05 was considered statistically significant.

## Results

### PCa patients with a PSA level < 4 ng/ml had more favorable tumor characteristics

Two hundred five thousand nine hundred thirteen prostate adenocarcinoma patients from January 1, 2010 to December 31, 2015 were identified in this study, and 24,054 (11.68%) of these patients were diagnosed with a PSA level < 4 ng/ml. As shown in Table 1, in the group of PSA level < 4 ng/ml, there were more patients aged< 65 (57.4%) compared with the patients aged≥65 (42.6%). Beides, the proportions of patients aged< 65 in the group of the PSA level of 4–10 ng/ml, 10–20 ng/ml and > 20 ng/ml were 49.8, 38.7 and 37.3%, respectively (*p* < 0.001). Interestingly, the PSA < 4 ng/ml group contained a significantly higher proportion of married men (69.3%) compared with groups of PSA level of 4–10 ng/ml, 10–20 ng/ml and > 20 ng/ml (p < 0.001). The Caucasian patients were more likely to have a lower PSA value, whereas the African-American patients had a higher PSA value. Furthermore, in the group of PSA level < 4 ng/ml, there were more patients in stage T2 than that in stage T1, which was opposite of the trend in the groups of PSA level of 4–10 ng/ml, 10–20 ng/ml and > 20 ng/ml. For the patients in stage T3 and T4, the proportion was found to increase with the increase of PSA value. In addition, the lymph node metastasis rate and metastatic stage at diagnosis were significantly higher in the patients with a PSA level > 10 ng/ml than that with a PSA level < 10 ng/ml. However, in the group of M1 stage, there were patients with a PSA level < 4 ng/ml (0.9%) compared with the patients with a PSA level of 4–10 ng/ml (0.5%). The patients with higher PSA values had higher Gleason grades (*p* < 0.001).

**Table 1:** Clinical characteristics of prostate cancer patients

Features	PSA				*P* value
	< 4	4–10	10–20	> 20	
Age					
< 65	13,817 (57.4)	63,034 (49.8)	12,405 (38.7)	8629 (37.3)	< 0.001
≥ 65	10,237 (42.6)	63,629 (50.2)	19,680 (61.3)	14,482 (62.7)	
Married status					
Married	16,661 (69.3)	84,764 (66.9)	19,799 (61.7)	12,530 (54.2)	< 0.001
Unmarried	4388 (18.2)	25,624 (20.2)	8142 (25.4)	7540 (32.6)	
Unknown	3005 (12.5)	16,275 (12.8)	4144 (12.9)	3041 (13.2)	
Insurance					
Insured	21,608 (89.8)	114,316 (90.3)	28,918 (90.1)	20,417 (88.3)	< 0.001
Uninsured	210 (0.9)	1578 (1.2)	606 (1.9)	830 (3.6)	
Unknown	2236 (9.3)	10,769 (8.5)	2561 (8.0)	1864 (8.1)	
Race					
Caucasian	19,460 (80.9)	96,796 (76.4)	23,140 (72.1)	15,639 (67.7)	< 0.001
African-American	3066 (12.7)	19,717 (15.6)	5875 (18.3)	5309 (23.0)	
Other	801 (3.3)	6578 (5.2)	2210 (6.9)	1619 (7.0)	
Unknown	727 (3.0)	3572 (2.8)	860 (2.7)	544 (2.4)	
T stage					
T1	8243 (34.3)	56,428 (44.5)	14,252 (44.4)	8916 (38.6)	< 0.001
T2	13,705 (57.0)	56,084 (44.3)	11,943 (37.2)	7968 (34.5)	
T3	1803 (7.5)	12,825 (10.1)	5305 (16.5)	4234 (18.3)	
T4	74 (0.3)	270 (0.2)	247 (0.8)	1158 (5.0)	
Unknown	229 (1.0)	1056 (0.8)	338 (1.1)	835 (3.6)	
N stage					
N0	23,083 (96.0)	121,322 (95.8)	29,675 (92.5)	17,942 (77.6)	< 0.001
N1	266 (1.1)	1565 (1.2)	1287 (4.0)	3374 (14.6)	
Unknown	705 (2.9)	3776 (3.0)	1123 (3.5)	1795 (7.8)	
M stage					
M0	23,847 (99.1)	125,996 (99.5)	31,213 (97.3)	16,996 (73.5)	< 0.001
M1	207 (0.9)	667 (0.5)	872 (2.7)	6115 (26.5)	
Gleason grade					
1	13,865 (57.6)	58,851 (46.5)	9635 (30.0)	2548 (11.0)	< 0.001
2	6020 (25.0)	37,375 (29.5)	8660 (27.0)	3712 (16.1)	
3	1922 (8.0)	15,144 (12.0)	5403 (16.8)	3649 (15.8)	
4	1261 (5.2)	9783 (7.7)	4604 (14.3)	5401 (23.4)	
5	986 (4.1)	5510 (4.4)	3783 (11.8)	7801 (33.8)	

Abbreviations: PSA, prostate-specific antigen

### Overall mortality of patients with a PSA level < 4 ng/ml was associated with age, marital status, race, Gleason grade, M stage and treatment approach

During the follow-up, cancer-specific death and overall death were detected in the PCa patients with a PSA level < 4 ng/ml. In this study, 488 cancer-specific deaths (2.03%) and 691 overall deaths (2.87%) were recorded. The multivariate Cox regression model revealed that cancer-specific mortality and overall mortality were associated with age, marital status, race, T stage, Gleason grade, and treatment approach (Table 2). The multivariate Cox regression model revealed that the M stage was also an important risk factor for overall mortality. As shown in Table 2, the patients who received RP had a significant prolongation of survival. The patients who underwent RP with or without RT had the lowest risk of mortality in the Cox model (vs. none, CCS: HR =0.346, 95% CI: 0.255–0.470, *P* < 0.001; OS: HR =0.316, 95% CI: 0.242–0.412, *P* < 0.001).

**Table 2:** Multivariate Cox regression analysis of prognostic factors for CSS and OS among primary prostate cancer patients with PSA < 4 ng/ml (diagnosed 2010–2015)

Variables	CSS			OS		
	HR	95% CI	*P* value	HR	95% CI	*P* value
Age						
< 65	1			1		
≥ 65	2.949	2.378–3.659	< 0.001	2.318	1.934–2.779	< 0.001
Married status						
Married	1			1		
Unmarried	1.784	1.451–2.194	< 0.001	1.626	1.366–1.936	< 0.001
Insurance						
Insured	1			1		
Uninsured	0.836	0.131–6.690	0.836	1.481	0.614–3.574	0.570
Race						
Caucasian	1			1		
African-American	1.361	1.069–1.733	0.012	1.260	1.018–1.558	0.033
Other	0.662	0.364–1.207	0.178	0.950	0.634–1.424	0.805
T stage						
T1	1			1		
T2	0.830	0.601–1.148	0.061	0.861	0.733–1.011	0.001
T3	2.249	1.423–3.553	0.003	1.007	0.733–1.384	0.440
T4	49.213	30.094–80.478	< 0.001	15.321	10.237–22.932	< 0.001
N stage						
N0	1			1		
N1	0.761	0.269–2.150	0.606	1.294	0.891–1.880	0.176
M stage						
M0	1			1		
M1	0.627	0.221–1.773	0.379	4.198	3.065–5.751	< 0.001
Gleason grade						
1	1			1		
2	1.385	1.111–1.727	0.004	1.383	1.120–1.709	0.003
3	1.017	0.699–1.481	0.929	1.135	0.806–1.597	0.469
4	1.770	1.240–2.525	0.002	2.960	2.256–3.883	< 0.001
5	2.402	1.648–3.499	< 0.001	6.059	4.725–7.770	< 0.001
Therapy						
No	1			1		
RP with or without RT	0.346	0.255–0.470	< 0.001	0.316	0.242–0.412	< 0.001
RT without RP	0.599	0.470–0.764	< 0.001	0.608	0.493–0.750	< 0.001

Abbreviations: PSA, prostate-specific antigen; CSS, cancer-specific survival; OS, overall survival; RP, radical prostatectomy; RT, radiation therapy (brachytherapy and/or external beam radiation); HR, hazard ratio; 95% CI, 95% confidence interval

**Fig. 1:**
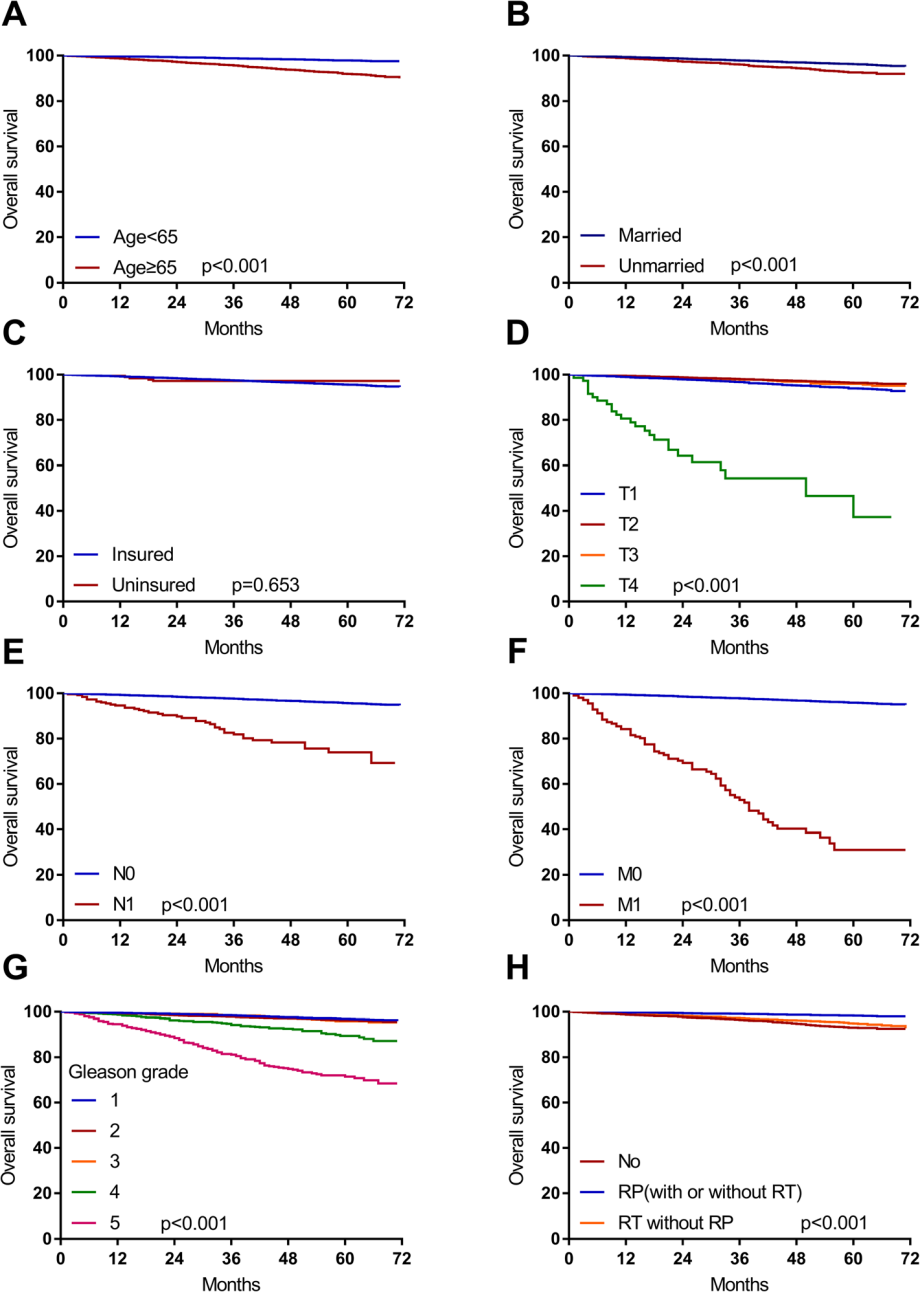
The overall survival curve of patients with a PSA level < 4 ng/ml associated with different factors. **a** Age < 65 and Age ≥ 65; (**b**) Marital status; (**c**) Insurance status; (**d**) T stage; (**e**) N stage; (**f**) M stage; (**g**) Gleason grade;(**h**) Treatment approach

The OS estimates were classified by age, marital status, insurance status, T stage, N stage, M stage, Gleason grade, and treatment approach (Fig. 1). The OS of patients aged≥65 years old was significantly shorter than that of patients aged<65 years old (*p* < 0.001). In the group of PSA < 4 ng/ml, the married patients had shorter OS compared with those who were unmarried (p < 0.001). However, there was no difference in OS between insured and uninsured patients (p>0.05). Among the patients with a PSA level < 4 ng/ml, the median survival of those in stage of M1 (median survival = 38 months, 95% CI: 31.9–44.1) or T4 (median survival = 50 months, 95% CI: 24.2–75.8) was the shortest. Furthermore, in the group of PSA < 4 ng/ml, patients in N1 stage and Gleason grade4 stage had shorter OS (*p* < 0.001). Thus, these results suggested patients with advanced TNM stage and high Gleason grade might had worse outcomes. Additionly, our results showed the 5-year OS rates of patients receiving no RP or radiotherapy (RT), receiving RP with or without RT and receiving RT without RP were 93.07, 98.30 and 94.89%, respectively (p < 0.001).

## Discussion

The serum PSA determination is the most commonly used method to detect PCa. Usually, the PSA level > 4 ng/ml is considered abnormal. Over the past few decades, a high frequency of PCa patients with a PSA level < 4 ng/ml has been reported [9]. Autopsy studies have shown that there was a high incidence of occult cancer in the prostate of men who die without being diagnosed with PCa [15]. Thus, the incidence of PCa patients with a PSA level < 4 ng/ml may be underestimated at initial diagnosis. Based on a large population analysis, we found that 11.68% of PCa patients were initially diagnosed with a PSA level < 4 ng/ml. Several novel findings have been identified in our study as follows.

First, the 24,054 PCa patients with a PSA level < 4 ng/ml had more favorable clinical characteristics than those with other PSA level(4–10 ng/ml, 10–20 ng/ml and > 20 ng/ml): the patients with a PSA level < 4 ng/ml were younger and diagnosed with lower T stages (T1–2), lower Gleason grades and lower lymph node metastasis rates. This is consistent with contemporary studies [10, 16, 17]. However, in our study, there were more cases in stage M1 among patients with a PSA level < 4 ng/ml compared with those with a PSA level of 4–10 ng/ml. PCa patients with a PSA level < 4 ng/ml should have undergone abnormal digital rectal examination or magnetic resonance imaging [18], so according to stage, the T1 stage patients comprised the lowest proportion of the patients among the different PSA level groups. Therefore, the PCa patients with a PSA level < 4 ng/ml may be biologically aggressive in some respects, and physicians should appropriately pay attention to prostate patients with a low PSA level. Besides, married patients and Caucasian patients were more likely to have lower PSA values; whereasAfrican-American men without PCa tended to have higher PSA values than Caucasian men without PCa [19].

Furthermore, several prognostic factors for PCa patients with a PSA level < 4 ng/ml, which were correlated with higher mortality risk, were found, including older age (≥65 years), unmarried status, African-American ethnicity, high T stage (T4), high M stage (M1), higher Gleason grade, and lack of surgery or radiotherapy. Our result suggested that the Gleason grading system had an affirmative predictive value in the prognosis of PCa patients with a PSA level < 4 ng/ml. Initial treatment of diagnosed male patients with PCa requires evaluation of clinical staging based on a digital rectal examination, the pretreatment serum PSA value, the Gleason score of a biopsy and the percentage of cancer involvement in the biopsy core. Though our study showed that patients could benefit fromsurgery and radiotherapy, and active surveillance could be considered as the preferred option for localized low-risk PCa patients [20–25]. Based on prognostic factors, the PCa patients with a PSA level < 4 ng/ml can receive a preliminary evaluation from physicians. For PCa patients with a PSA level < 4 ng/ml, risk stratification (high Gleason grade and T stage), age, life expectancy, comorbidities and patient preferences should be considered in the choice of treatment strategies.

To our knowledge, this is the first large-sample-size population-based study focused on PCa patients with a PSA level < 4 ng/ml. Moreover, our study is based on a comprehensive design, including analysis of tumor characteristics, patient treatments, and survival outcomes. However, there were several limitations in our study. First, information on prostate volume, family history, body mass index or biochemical recurrence was not available in the SEER database. Similarly, the information on RT radiation dosage was unavailable. Last but not least, the reasons for such patients to visit doctors were not available. Besides, the data of PSA from SEER database are limited. We will enrich the conclusions drawn in our study through other data sources in future research, and make more comprehensive and in-depth analysis and summary of tumor characteristics, treatments, and survival outcomes for patients with PSA < 4 ng / ml.

## Conclusion

In conclusion, PCa patients with a PSA level < 4 ng/ml have more favorable tumor characteristics at diagnosis and receive more benefit from active treatment. However, we should pay more attention to the subset of these patients with advanced TNM stage and high Gleason grade.

## Data Availability

The datasets generated and analyzed during the current study are available from the corresponding author on reasonable request.

## Abbreviations

PCa: prostate cancer
SEER: Surveillance, Epidemiology and End Results
PSA: prostate-specific antigen
RP: radical prostatectomy
BT: brachytherapy
EBRT: external beam radiation
RT: radiotherapy
CCS: cancer-specific survival
OS: overall survival
M1: high M stage
